# A risk prediction nomogram for resistant hypertension in patients with obstructive sleep apnea

**DOI:** 10.1038/s41598-024-56629-7

**Published:** 2024-03-13

**Authors:** Hongze Lin, Chen Zhou, Jiaying Li, Xiuqin Ma, Yan Yang, Taofeng Zhu

**Affiliations:** 1 Department of General Practice, The Yixing Hospital affiliated to Jiangsu University, Yixing, 214200 China; 2 Department of Respiratory and Critical Care Medicine, Yixing Hospital affiliated to Jiangsu University, Yixing, 214200 China

**Keywords:** Obstructive sleep apnea, Resistant hypertension, Risk factors, Nomogram, Model, Medical research, Risk factors

## Abstract

Patients with obstructive sleep apnea (OSA) are liable to have resistant hypertension (RH) associated with unfavorable cardiovascular events. It is of necessity to predict OSA patients who are susceptible to resistant hypertension. Hence, we conducted a retrospective study based on the clinical records of OSA patients admitted to Yixing Hospital Affiliated to Jiangsu University from January 2018 to December 2022. According to different time periods, patients diagnosed between January 2018 and December 2021 were included in the training set (n = 539) for modeling, and those diagnosed between January 2022 and December 2022 were enrolled into the validation set (n = 259) for further assessment. The incidence of RH in the training set and external validation set was comparable (*P* = 0.396). The related clinical data of patients enrolled were collected and analyzed through univariate analysis and least absolute shrinkage and selection operator (LASSO) logistic regression analysis to identify independent risk factors and construct a nomogram. Finally, five variables were confirmed as independent risk factors for OSA patients with RH, including smoking, heart disease, neck circumference, AHI and T90. The nomogram established on the basis of variables above was shown to have good discrimination and calibration in both the training set and validation set. Decision curve analysis indicated that the nomogram was useful for a majority of OSA patients. Therefore, our nomogram might be useful to identify OSA patients at high risk of developing RH and facilitate the individualized management of OSA patients in clinical practice.

## Introduction

Obstructive sleep apnea (OSA) is characterized by repeated collapse of the upper airway during sleep, resulting in inadequate ventilation, intermittent hypoxia, hypercapnia and sleep structure disorder^1^. The main manifestations of patients with OSA include sleep snoring, apnea, daytime sleepiness, lack of concentration and poor quality of life. With the increasing prevalence of obesity, the incidence of OSA patients is increasing. Nowadays, OSA has become a growing public health problem since about 1 million people around the world are at risk of it^2^. Importantly, OSA has been demonstrated as an independent risk factor for cardiovascular and cerebrovascular diseases, including ischemic heart disease, hypertension and stoke, which contributes to increased morbidity and all-cause mortality^3–6^.

Resistant hypertension (RH) is a condition that blood pressure is continuously higher than 140/90 mmHg, though three or more antihypertensive drugs including a diuretic are provided. Collective evidence suggests that there is a significant correlation between OSA and RH^7–9^. The prevalence of OSA in RH patients is 70–83%, and the risk of RH in patients with OSA increases by 116%^9,10^. OSA is the most common disease associated with RH and has been confirmed as an independent risk factor for RH^11^. In addition, OSA patients with RH are prone to adverse cardiovascular consequences, which seriously endanger the health and the life of patients^12^. Hence, investigation of relevant risk factors and establishment of a nomogram to determine OSA patients at high risk of developing RH would be clinically useful to early diagnosis and effective treatment. Accurate assessment of the risk of RH in OSA patients is of importance for timely prevention, early diagnosis, effective treatment. However, currently, few studies focus on identifying risk factors of RH in patients with OSA, and there is no relevant predictive model.

Least absolute shrinkage and selection operator (LASSO) is a machine learning method, which can continuously shrink the coefficients of variables by constructing a penalty function to avoid collinearity and overfitting, thereby simplifying the model^13^. The effect of LASSO analysis in variable selection is better than traditional methods, such as stepwise regression, principal component regression, ridge regression and partial least squares. Recently, LASSO has been frequently used for variable selection before model construction^14^. Logistic regression is a generalized linear regression analysis model, which is often used to establish model of predicting the probability of disease or event in combination with LASSO analysis^15–18^.

In this study, we aimed to determine the risk factors and construct a useful nomogram to predict the risk of RH in OSA patients through LASSO logistic regression analysis, which could help clinicians to ascertain the high-risk population of RH in OSA patients and carry out precise prevention.

## Methods

### Data source and study population

We performed a retrospective single-center study based on the clinical records of OSA patients admitted to Yixing Hospital Affiliated to Jiangsu University from January 2018 to December 2022. OSA patients diagnosed between January 2018 and December 2021 were divided into training set for modeling, and the subjects diagnosed between January 2022 and December 2022 were divided into external validation set. This study was approved by the Ethics Committee of Yixing Hospital Affiliated to Jiangsu University (approval no. 2022WEN083). Informed consent was obtained from all subjects and/or their legal guardian(s). Patients filled with following characteristics were included: (1) age ≥ 18 years old; (2) meeting the diagnostic criteria of OSA referring to clinical guideline for Adult Obstructive Sleep Apnea of 2017^19^; (3) being able to complete the questionnaire independently, completely and accurately; (4) no history of OSA treatment; (5) signing the consent form and cooperating to complete the study. However, the exclusion criteria were as follows: (1) hypertension secondary to endocrine causes (pheochromocytoma, Conn’s disease, Cushing’s syndrome, hyperparathyroidism), renal artery stenosis, coarctation of the aorta, and drug induction; (2) severe cardiopulmonary diseases and malignant tumors; (3) central or mixed sleep apnea; (4) previous mental illness or intolerance to polysomnography (PSG) monitoring; (5) incomplete clinical data and PSG monitoring data. The diagnostic criteria of RH were specified by the global initiative for hypertension^20^.

### Risk factors

Baseline information of the enrolled patients was recorded, including gender, age, body mass index (BMI), neck circumference, waist circumference, smoking, alcohol drinking, family history of hypertension, and comorbidities composed of RH, diabetes and heart disease (coronary heart disease, atrial fibrillation and myocardial infraction). The Epworth sleepiness scale (ESS) was also filled to assess daytime sleepiness. All patients included in this study underwent overnight PSG monitoring using SOMNOlab 2 model machine (Wanman, Germany), and the collected indicators included total sleep time, apnea–hypopnea index (AHI), oxygen desaturation index (ODI), percentage of total time with oxygen saturation level < 90% (T90), lowest arterial oxygen saturation (LSaO2) and mean arterial oxygen saturation (MSaO2). In addition, fasting venous blood (FBG) of all subjects was collected within 24 h before discharge using SIEMENS ADVIA 1200 automatic biochemical analyzer (SIEMENS, Germany) to obtain data on total cholesterol, triglyceride, high density lipoprotein and low density lipoprotein. These variables were considered as potential risk factors for further selection of predictors.

### Statistical analysis

Statistical analysis was conducted using IBM SPSS Statistics (version 27.0.1, Chicago, USA) and R software 4.2.2 (R foundation for Statistical Computing, Vienna, Austria). Data were described as mean (standard deviation [SD]) or median (interquartile range [IQR]) for continuous variables and number (percentage) for categorical variables as appropriate. In order to compare the differences in baseline clinical characteristics between the training set and the validation set, and between patients with RH and without RH in training set, bivariate analysis was performed by chi-squared test or Fisher’s exact test for categorical variables, t test for normal distribution continuous variables, and Mann–Whitney U test for non-normal distribution variables. The variables with *P* < 0.05 in the univariate analysis of the training set were substituted into LASSO analysis for further variable screening. According to the results of LASSO analysis, multivariate logistic regression analysis was implemented to further clarify independent risk factors of RH in OSA patients. The results were reported as odds ratios (ORs) and 95% confidence intervals (95%CIs). We then constructed a nomogram based on independent risk factors to predict the risk of developing RH in patients with OSA using package ‘rms’ in R. Patients in the external validation set were used for assessing the discrimination and calibration of the predictive nomogram. The discriminative performance was evaluated using the area under receiver operating characteristic (ROC) curve (AUC), known as the c-statistic. Calibration of the model was assessed by comparison of the predicted and observed probability of RH in patients with OSA. The fitting degree of the scoring model was measured by Hosmer–Lemeshow goodness of fit test. Decision curve analysis was performed to evaluate the clinical utility of the nomogram, using the package ‘dcurves’ in R. Unless otherwise stated, a two-tailed *P* value < 0.05 was considered statistically significant. All methods were carried out in accordance with relevant guidelines and regulations.

### Ethics declarations

The study protocol was approved by the Ethics Committee of Yixing Hospital Affiliated to Jiangsu University (approval no. 2022WEN083). All the patients enrolled were informed consent.

## Results

### Characteristics of study patients

**Figure 1:**
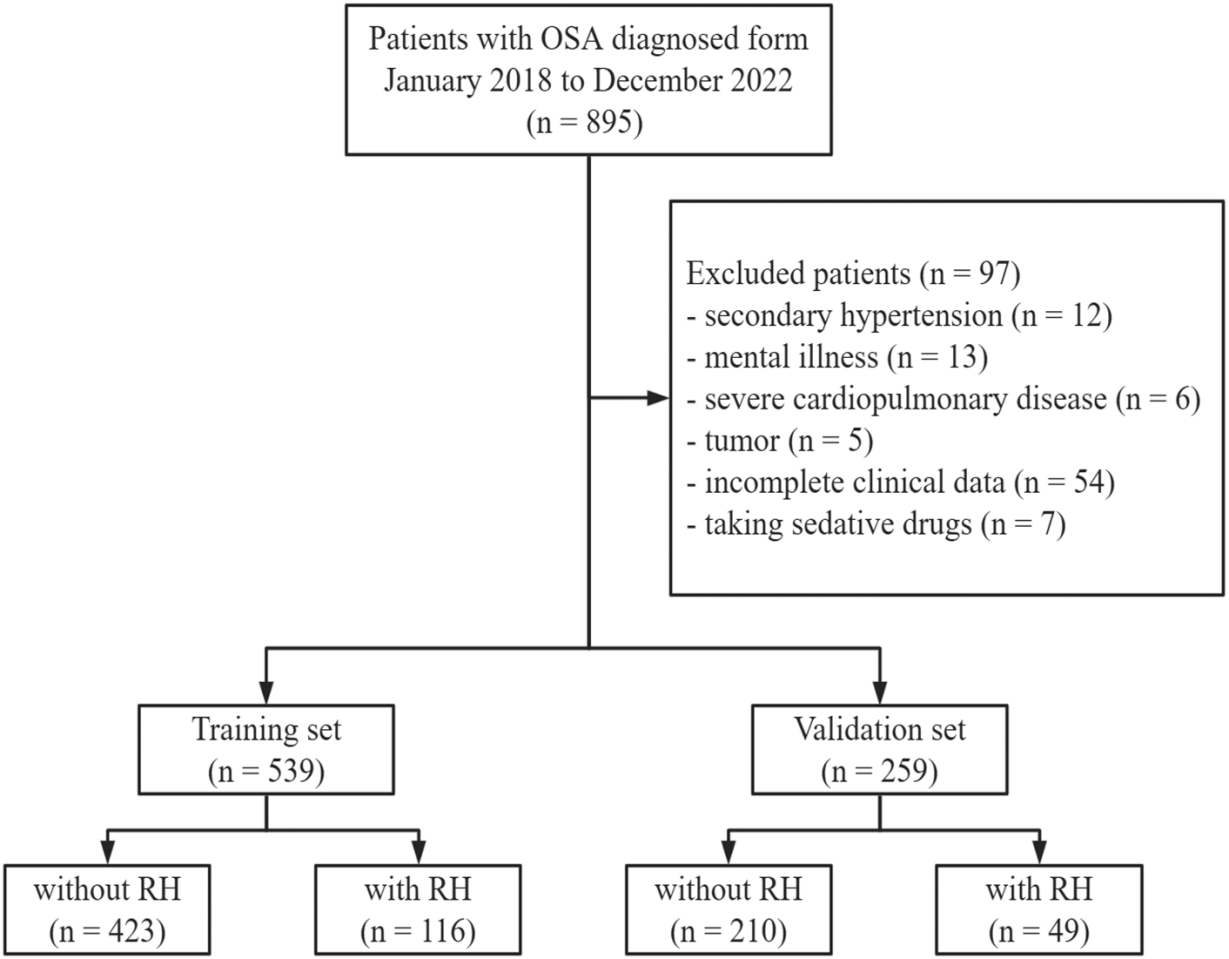
Study population screening. *OSA*, obstructive sleep apnea; *RH*, resistant hypertension.

A total of 895 OSA patients diagnosed from January 2018 to December 2022 were originally screened. 97 patients who met the exclusion criteria were excluded for secondary hypertension (n = 12), mental illness (n = 13), severe cardiopulmonary disease (n = 6), tumor (n = 5), incomplete clinical data (n = 54) and taking sedative drugs (n = 7). Finally, 539 patients were assigned to the training set, and 262 patients were included to the validation set (Fig. 1). The baseline characteristics of patients in the training set and validation set and the comparison between two sets were shown in Table 1. The incidence of RH in the training set (21.5%) and external validation set (18.9%) was comparable (*P* = 0.396). There was no significant difference in most of the characteristics between the two sets.

**Table 1:** Basic characteristics in the training set and validation set.

Characteristic	Training set (n = 539)	Validation set (n = 259)
Sex		
Male	437 (81.1)	210 (81.1)
Age (year)	52.26 (13.58)	54.23 (12.67)
BMI (kg/m^2^)	27.30 (4.01)	27.16 (4.06)
Smoke	198 (36.7)	90 (34.7)
Alcohol	65 (12.1)	33 (12.7)
Family history of hypertension	169 (31.4)	77 (29.7)
Heart disease	71 (13.2)	38 (14.7)
Diabetes	101 (18.7)	45 (17.4)
Resistant hypertension	116 (21.5)	49 (18.9)
Neck circumference (cm)	36.13 (2.05)	36.22 (2.2)
Waist circumference (cm)	95.11 (5.10)	95.76 (7.17)
ESS	10.72 (3.17)	10.81 (2.14)
Total sleep time (h)	7.41 (1.88)	7.7 (1.66)
AHI	29.57 (17.87)	27.31 (16.27)
T90 (%)	1.88 (7.53)	1.59 (6.61)
ODI	20.32 (18.91)	18.14 (17.38)
LSaO2 (%)	76.36 (10.67)	77.61 (13.18)
MSaO2 (%)	94.87 (4.53)	94.96 (2.68)
TC (mmol/L)	4.31 (0.99)	4.34 (1.02)
Triglyceride (mmol/L)	2.00 (1.71)	2.01 (1.15)
HDL (mmol/L)	1.11 (0.25)	1.08 (0.22)
LDL (mmol/L)	2.57 (0.76)	2.56 (0.71)
FBG (mmol/L)	5.77 (1.56)	5.86 (1.85)

*BMI*, body mass index; *ESS*, Epworth sleepiness scale; *AHI*, apnea–hypopnea index; *T90*, percentage of total time with oxygen saturation level < 90%; *ODI*, oxygen desaturation index; *LSaO2*, lowest arterial oxygen saturation; *MSaO2*, mean arterial oxygen saturation; *TC*, total cholesterol; *HDL*, high density lipoprotein; *LDL*, low density lipoprotein; *FBG*, fasting blood glucose.

### Nomogram construction

**Figure 2:**
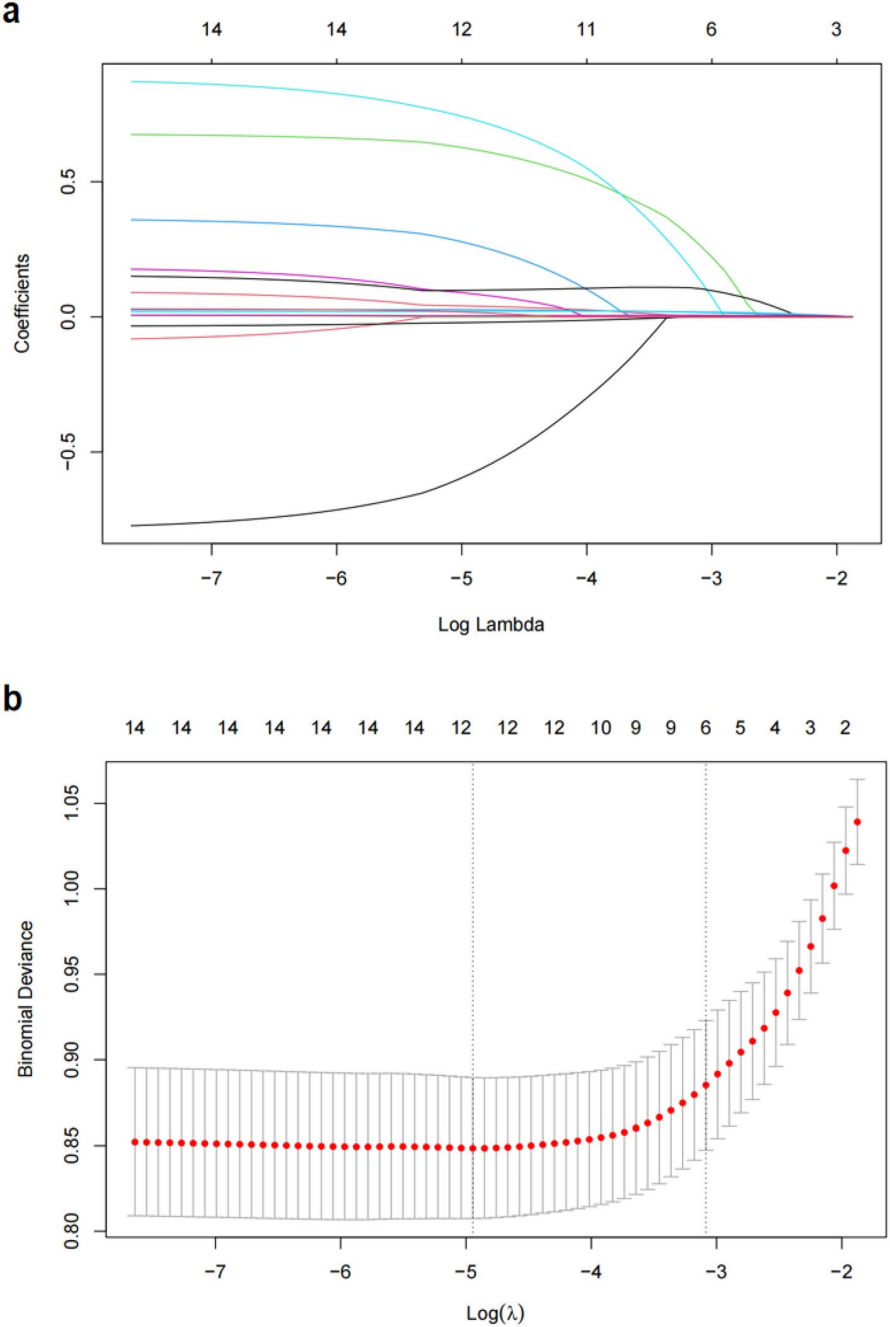
LASSO regression analysis. (**a**) LASSO coefficient profiles of the 14 variables. As the value of λ decreased, the later the coefficient was compressed, the more important the variable was. (**b**) Cross-validation results. The value between the two dashed lines is the range of positive and negative standard deviations of log(λ). The dashed line on the right reveals that when the fitting effect of the model is good, the number of variables contained is the least. Finally, six variables were selected when log(λ) = 0.045.

**Figure 3:**
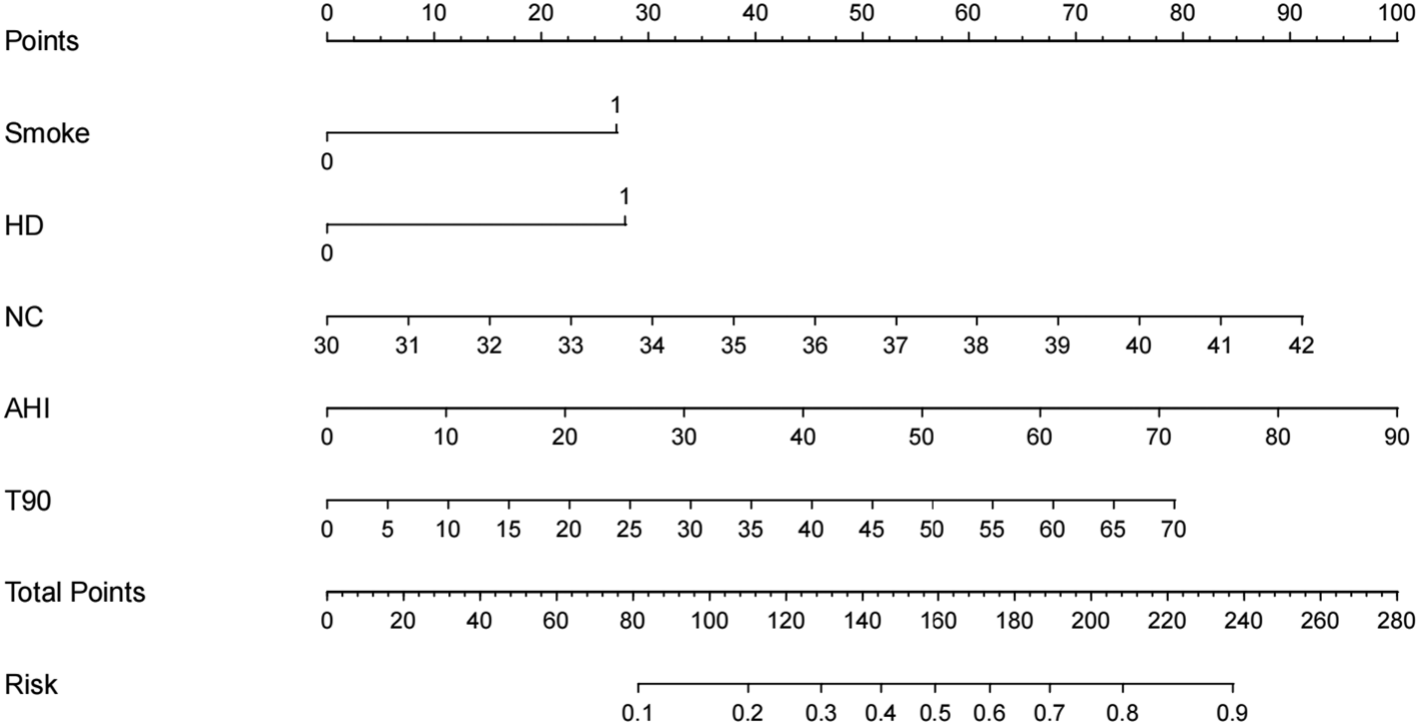
The nomogram of predicting the risk of RH for individual patient with OSA. *RH*, resistant hypertension; *OSA*, obstructive sleep apnea; *HD*, heart disease; *NC*, neck circumference; *AHI*, apnea–hypopnea index; *T90*, percentage of total time with oxygen saturation level < 90%.

According to the results of initial screening using univariate analysis, fourteen variables had significant differences between OSA patients with RH and OSA patients without RH in the training set, including male gender, BMI, smoking, family history of hypertension, comorbidities of heart disease and diabetes, neck circumference, waist circumference, ESS, AHI, T90, ODI, MSaO2 and FBG (Table 2). These variables were further analyzed through LASSO regression analysis, and six variables were selected for multivariate logistic regression analysis (Fig. 2a, b). In the multivariate logistic regression analysis, smoking, heart disease, neck circumference, AHI, T90 were demonstrated as significant independent risk factors for RH in OSA patients. The detailed OR values and 95%CI in the multivariate analysis were listed in Table S1. We developed a risk prediction model based on the above five parameters through logistic regression analysis (Table 3). In order to facilitate the assessment of the individual risk of RH in OSA patients, a nomogram was constructed according to the contribution weight of each factor in the risk model (Fig. 3). In the nomogram, the values of each factor have corresponding points on the scale axis. The total score for each patient can be easily calculated by adding up single points of each factor. By projecting the total score to the bottom of the total subscale, the risk of developing RH in each patient with OSA could be estimated.

**Table 2:** Comparison of baseline characteristics between OSA patients with and without RH in the training set.

Characteristic	OSA patients		P
	Without RH (n = 423)	With RH (n = 116)	
Sex			
Male	331 (78.3%)	106 (91.4%)	0.001
Age (year)	52.15 (13.71)	52.66 (13.17)	0.719
BMI (kg/m^2^)	26.83 (3.81)	28.99 (4.26)	< 0.001
Smoke	135 (31.9%)	63 (54.3%)	< 0.001
Alcohol	52 (12.3%)	13 (11.2%)	0.75
Family history of hypertension	113 (26.7%)	56 (48.3%)	< 0.001
Heart disease	44 (10.4%)	27 (23.3%)	< 0.001
Diabetes	68 (16.1%)	33 (28.4%)	0.002
Neck circumference (cm)	35.81 (1.90)	37.32 (2.12)	< 0.001
Waist circumference (cm)	94.46 (4.98)	97.47 (4.85)	< 0.001
ESS	10.21 (2.99)	12.61 (3.1)	< 0.001
Total sleep time (h)	7.47 (1.86)	7.18 (1.96)	0.148
AHI	26.26 (15.78)	41.61 (19.84)	< 0.001
T90 (%)	1.16 (4.59)	9.35 (22.19)	< 0.001
ODI	11.73 (20.18)	28.67 (34.07)	< 0.001
LSaO2 (%)	76.79 (11.16)	74.79 (8.53)	0.075
MSaO2 (%)	95.41 (2.12)	92.88 (8.62)	< 0.001
TC (mmol/L)	4.29 (0.98)	4.41 (1.01)	0.253
Triglyceride (mmol/L)	1.98 (1.69)	2.08 (1.81)	0.572
HDL (mmol/L)	1.12 (0.25)	1.08 (0.21)	0.077
LDL (mmol/L)	2.55 (0.77)	2.64 (0.72)	0.241
FBG (mmol/L)	5.68 (1.42)	6.11 (1.94)	0.009

*RH*, resistant hypertension; *BMI*, body mass index; *ESS*, Epworth sleepiness scale; *AHI*, apnea–hypopnea index; *T90*, percentage of total time with oxygen saturation level < 90%; *ODI*, oxygen desaturation index; *LSaO2*, lowest arterial oxygen saturation; *MSaO2*, mean arterial oxygen saturation; *TC*, total cholesterol; *HDL*, high density lipoprotein; *LDL*, low density lipoprotein; *FBG*, fasting blood glucose.

**Table 3:** Multivariate logistic regression for RH in the training set.

Variable	β	SE	P	OR (95%*CI*)
Smoke	0.762	0.247	0.002	2.144 (1.320, 3.481)
Heart disease	0.785	0.320	0.014	2.193 (1.170, 4.111)
Neck circumference	0.214	0.065	< 0.001	1.239 (1.091, 1.407)
AHI	0.031	0.007	< 0.001	1.032 (1.017, 1.047)
T90	0.031	0.009	< 0.001	1.032 (1.014, 1.051)

*AHI*, apnea–hypopnea index; *T90*, percentage of total time with oxygen saturation level < 90%; *SE*, standard error; *OR*, odds ratio; *CI*, confidence interval.

### Nomogram assessment

**Figure 4:**
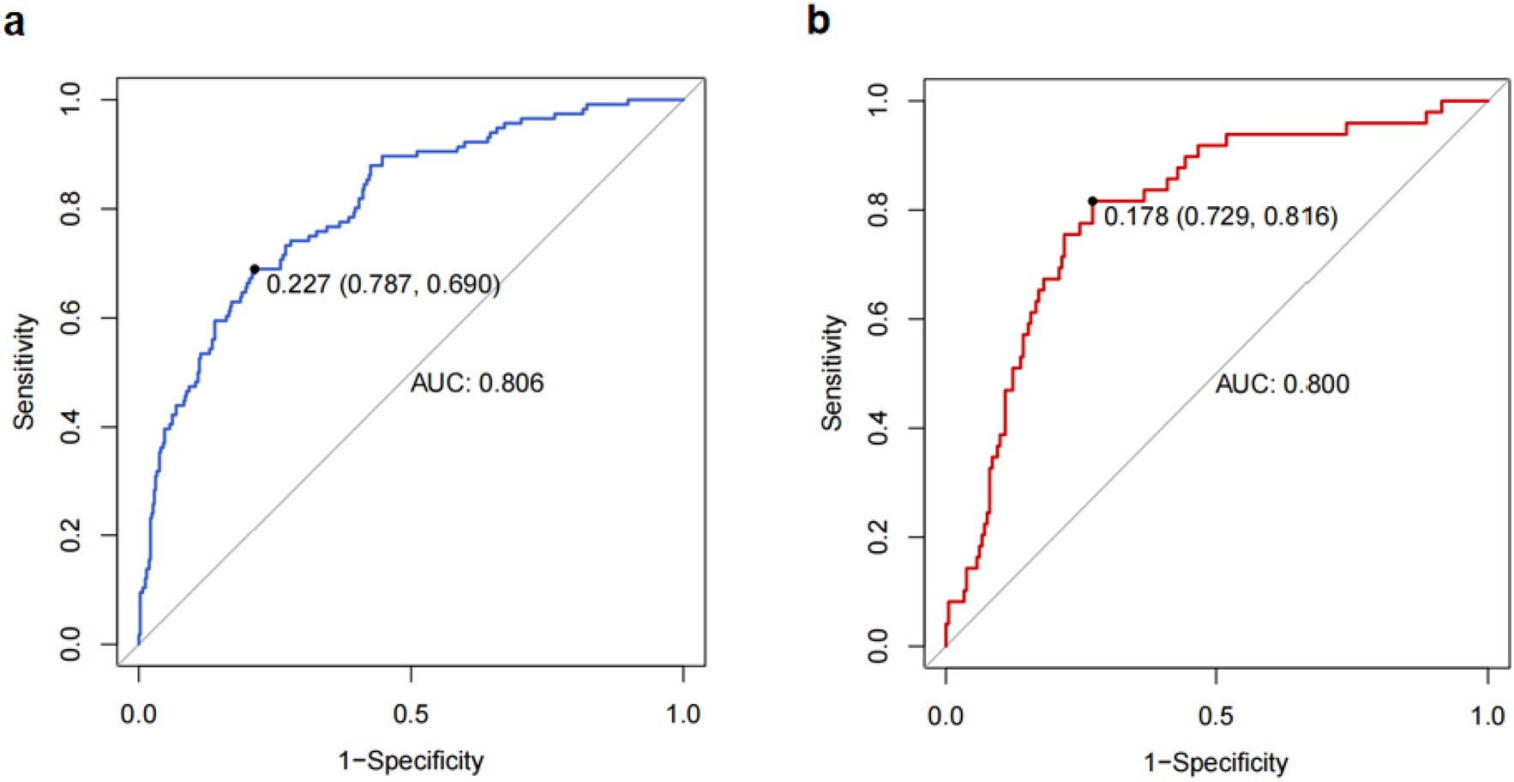
Receiver operating characteristic (ROC) curve analysis. (**a**) ROC curve of the prediction nomogram in the training set. (**b**) ROC curve of the prediction nomogram in the validation set. *AUC*, area under the curve.

**Figure 5:**
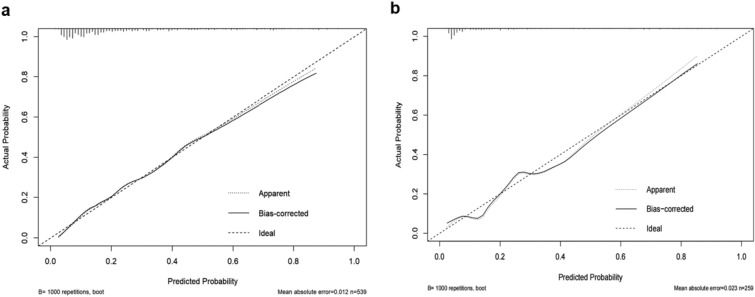
(**a**) Calibration curve of the nomogram in the training set. (**b**) Calibration curve of the nomogram in the validation set.

The c-statistic was 0.806 (95%CI: 0.761–0.851) in the training set (Fig. 4a) and 0.800 (95%CI: 0.732–0.867) in the validation set (Fig. 4b). The calibration curves demonstrated that the probability predicted by the nomogram was relatively consistent with the actual observed probability in the training set (Fig. 5a) and validation set (Fig. 5b). The *P* values of Hosmer–Lemeshow test in the training set and validation set were both more than 0.05, which were 0.618 and 0.525, respectively, indicating a favorable calibration of the risk prediction model. In conclusion, the nomogram for RH had a decent discriminative and calibrating performance in both the training set and validation set.

**Figure 6:**
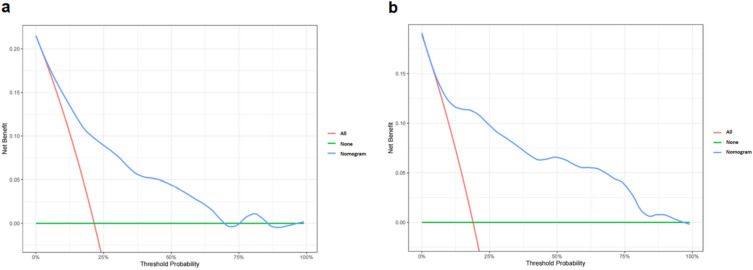
(**a**) Decision curve analysis of the nomogram in the training set. (**b**) Decision curve analysis of the model in the validation set. The red line (All) is the net benefit of RH treatment for all OSA patients. The green line (None) represents the net benefit of not providing RH treatment for OSA patients. The blue curve indicates the net benefit provided by the nomogram.

Decision curve analysis was conducted to evaluate the practicality and clinical application value of the nomogram, which showed that the majority of the threshold probabilities had great net benefit in both the training set (Fig. 6a) and validation set (Fig. 6b). In the decision curve analysis, the nomogram achieved a positive net benefit in the training set and validation set when predicted risk thresholds was between 19% and 81%, suggesting that the nomogram established could be alternative tool for clinical practice.

## Discussion

Nomogram is a fast and cost-effective method that is easy to implement in clinical practice. This retrospective study is the first study to develop and validated a risk prediction nomogram to identify OSA patients at high risk of RH. The nomogram performed a good discrimination and calibration in the training set, which was confirmed in the validation set. Decision curve analysis supported that the nomogram could provide an ideal reference value for clinical practice. Our nomogram consisted of five clinical characteristics, including smoking, heart disease, neck circumference, AHI and T90. In the nomogram, the individual risk score of RH could be calculated manually by collecting related medical history and routine parameters tested in the laboratory.

OSA is the most common comorbidity and independent risk factor of RH. Epidemiological data reported that the prevalence of OSA in patients with RH was as high as 70–83%^21,22^. Hou et al.^23^ found a significant association between OSA and RH with a pooled OR value of 2.842 (95%CI: 1.703–3.980), indicating that the risk of RH in OSA patients was 1.842 times higher than that in non-OSA individuals. However, the prevalence of RH in OSA patients has not been directly investigated. The prevalence of RH was 14.7% in 3.2 million patients with hypertension^24^. In the present study, the prevalence of RH in patients with OSA was as high as 21.5% in the training set and 18.9% in the validation set. The reason for the relatively high prevalence of RH may be that all the subjects included in this study were OSA patients, resulting in a higher proportion of RH in the enrolled population than in the general population.

In comparison with patients with controlled blood pressure, associated risk factors of RH are older age, obesity, diabetes, and silent target organ damage (left ventricular hypertrophy or chronic kidney disease)^25,26^. However, there are few studies on the risk factors of RH in patients with OSA. In the present study, the LASSO-multivariate logistic regression analysis demonstrated that smoking, heart disease, neck circumference, AHI and T90 were independent risk factors of RH in OSA patients. Ahmed et al.^22^ found that smoking and obesity were associated with increased risk for resistant hypertension in OSA patients. Smoking plays an important role in increasing blood pressure via activating sympathetic nerve system and interfering with the effects induced by certain antihypertensive drugs^27^. Neck circumference is commonly used to measure the degree of obesity in individuals, which has been demonstrated as independent risk factor for OSA with hypertension^28^. Increased neck circumference means raised fact accumulation around the upper respiratory tract, contributing to upper respiratory tract stenosis and intermittent hypoxia, subsequently activating the renin-angiotensin aldosterone system, thereby leading to water and sodium retention and making blood pressure difficult to control^29,30^. In this study, heart diseases consisted of coronary heart disease, atrial fibrillation and myocardial infraction. OSA is quite common in patients with heart disease and interact with heart disease to lead to unfavorable cardiovascular events^31^. Zhu et al.^32^ determined that the prevalence of resistant hypertension was relatively high in patients with heart disease. AHI and T90 are important indicators for evaluating intermittent hypoxia in OSA patients. Shi et al.^33^ investigated that T90 was a risk factor for hypertension related with OSA. Mechanically, intermittent hypoxia in patients with OSA can also cause excessive activation of the renin-angiotensin aldosterone system and vascular endothelial dysfunction, resulting in elevated blood pressure without control and a significant increase in the risk of RH^34^.

The consensus on resistant hypertension has identified the causal relationship between OSA and RH as relevant^35^. OSA is characterized by brain microarousal, intrathoracic pressure changes and intermittent hypoxia, which in turn triggers adverse biological processes such as oxidative stress, sympathetic activation, inflammation, hypercoagulability, endothelial dysfunction and metabolic disorders, making OSA patients susceptible to hypertension^36^. Prabhakar et al.^34^ found that intermittent hypoxia increased the levels of reactive oxygen species (ROS) by up-regulating hypoxia-inducible factor (HIF) -1α and reducing HIF-2α protein levels, which activated chemoreflex and inhibiting baroreflex, thereby resulting in hypertension-related sympathetic activation. In addition, patients with resistant hypertension have high aldosterone levels, and fluid retention associated with elevated aldosterone aggravates OSA by promoting fluid movement to the surrounding tissues, especially the neck, leading to secondary pharyngeal edema and an increased tendency of airway collapse during sleep^37^.

Hypertension is one of the most important risk factors for cardiovascular disease related to death, especially stroke, ischemic heart disease, congestive heart failure, aortic aneurysm and peripheral arterial disease^38,39^. RH patients suffer a higher risk of end-organ damage contributing to death in comparison to those with controlled blood pressure, including carotid stenosis, retinopathy, left ventricular hypertrophy and heart failure, myocardial infraction, stroke, impaired renal function^40,41^. In addition to its clinical importance, RH also bears a considerable public health, economic and social burden due to the cost of treatment and related disability and premature death. Hence, early detection of RH in OSA patients and timely intervention are of great significance to avoid adverse consequence. For the issue how to treat with OSA patients at high risk of developing RH, several studies suggested that continuous positive airway pressure (CPAP) treatment which is the gold standard treatment for OSA played an important role in lowering blood pressure^42–46^. Labarca et al.^7^ conducted a systematic review and meta-analysis and proved that CPAP benefited patients with RH and OSA by reducing blood pressure, especially nocturnal blood pressure. In a randomized controlled trail, CPAP treatment for 12 weeks reduced 24-h mean blood pressure and diastolic blood pressure and improved nocturnal blood pressure pattern in OSA patients with RH^43^. Bocoum et al.^45^ indicated that long-term CPAP for more than 4 h/night could reduce the all-cause mortality of RH patients. Overall, CPAP treatment seems to be an effective supplement to antihypertensive drugs for OSA patients with RH. Further studies on whether CPAP treatment can help prevent the occurrence of RH in patients with OSA are vital.

This study also had some limitations. First, this is a single-center cross-sectional study with retrospective design and selection bias. The causal relationship between OSA and RH has not been interpreted fully in this study. Prospective multicenter cohort studies with a larger sample size are needed to further prove the accuracy and reliability of the nomogram. Additionally, only one night of PSG monitoring was conducted and the sleep data of OSA patients might fluctuate, which might make the obtained data inaccurate. Multiple monitoring is needed to improve the authenticity of the data.

## Conclusion

Currently, most studies focus on the relationship between OSA and RH, but few studies pay attention to the risk factors of RH in OSA patients. In this study, we identified five independent risk factors for RH in patients with OSA and established a corresponding prediction nomogram for the first time. The nomogram is considered to be a reliable tool for predicting individual risk of RH in OSA patients, which could avail to early detection and precise intervention for susceptible patients. The follow-up multi-center prospective study are needed to validate the accuracy of the nomogram in clinical practice.

### Supplementary Information

Supplementary Table S1.

## Data Availability

The datasets used and/or analyzed during the current study are available from the corresponding author on reasonable request.
