# Supplementary material for: A risk prediction nomogram for resistant hypertension in patients with obstructive sleep apnea
Source: Sci Rep. 2024 Mar 13;14:6127. doi: 10.1038/s41598-024-56629-7 (PMC10937983; doi:10.1038/s41598-024-56629-7)
Supplement: Supplementary file 1 — Supplementary Table S1. [file 41598_2024_56629_MOESM1_ESM.docx]

**Table S1** Multivariate logistic regression for RH in training set.

| **Variable** | **β** | **SE** | **P** | **OR (95%*CI*)** |
| --- | --- | --- | --- | --- |
| Smoke | 0.777 | 0.248 | 0.001 | 2.176 (1.337, 3.541) |
| Heart disease | 0.804 | 0.321 | 0.012 | 2.236 (1.191, 4.198) |
| Neck circumference | 0.201 | 0.066 | 0.002 | 1.223 (1.073, 1.395) |
| AHI | 0.028 | 0.008 | < 0.001 | 1.029 (1.013, 1.045) |
| T90 | 0.024 | 0.012 | 0.047 | 1.025 (1.000, 1.051) |
| ODI | 0.008 | 0.010 | 0.413 | 1.009 (0.988, 1.029) |

***RH*, resistant hypertension; *AHI*, apnea-hypopnea index; *T90*, percentage of total time with oxygen saturation level < 90%; *SE*, standard error; *OR*, odds ratio; *CI*, confidence interval.**
